# Microbiome Composition and *Borrelia* Detection in *Ixodes scapularis* Ticks at the Northwestern Edge of Their Range

**DOI:** 10.3390/tropicalmed5040173

**Published:** 2020-11-18

**Authors:** Janet L. H. Sperling, Daniel Fitzgerald, Felix A. H. Sperling, Katharine E. Magor

**Affiliations:** 1 Department of Biological Sciences, University of Alberta, Edmonton, AB T6G 2E9, Canada; felix.sperling@ualberta.ca (F.A.H.S.); kmagor@ualberta.ca (K.E.M.); 2 Alberta Agriculture and Forestry, Agri-Food Laboratories, 6909-116 Street, Edmonton, AB T6H 4P2, Canada; dan.fitzgerald@ualberta.ca

**Keywords:** microbiome, Lyme disease, *Borrelia*, microbial diversity, *Ixodes*

## Abstract

Lyme disease-causing *Borrelia burgdorferi* has been reported in 10–19% of *Ixodes* ticks from Alberta, Canada, where the tick vector *Ixodes scapularis* is at the northwestern edge of its range. However, the presence of *Borrelia* has not been verified independently, and the bacterial microbiome of these ticks has not been described. We performed 16S rRNA bacterial surveys on female *I. scapularis* from Alberta that were previously qPCR-tested in a Lyme disease surveillance program. Both 16S and qPCR methods were concordant for the presence of *Borrelia.* The 16S studies also provided a profile of associated bacteria that showed the microbiome of *I. scapularis* in Alberta was similar to other areas of North America. Ticks that were qPCR-positive for *Borrelia* had significantly greater bacterial diversity than *Borrelia*-negative ticks, on the basis of generalized linear model testing. This study adds value to ongoing tick surveillance and is a foundation for deeper understanding of tick microbial ecology and disease transmission in a region where *I. scapularis* range expansion, induced by climate and land use changes, is likely to have increasing public health implications.

## 1. Introduction

Accurate characterization of the microbial communities that are associated with ticks is essential to understanding both the ticks and the diseases they transmit [1,2,3]. The most diverse and well-studied components of tick microbiomes are the bacteria [4], with different bacteria often interacting by interspecific and strain-level competitive displacement [5,6,7]. The assembly of such a microbial community can be affected by the immune responses of a tick to previously acquired microbes [8] and host-associated responses to tick salivary proteins [9]. The impacts of these bacteria on ticks are varied and can include positive effects such as increased reproductive output [4,10], overwintering success [11,12], or egg survival [13]. Although nutritional endosymbionts are the most abundant bacteria found in ticks, soil-associated bacteria are usually the most diverse [14,15], and even minor microbiome members can be critical during off-host periods in the lifecycle of ticks [16].

Since ticks often carry bacteria that are pathogenic to their vertebrate hosts, characterization of their microbial assemblages has historically focussed on only a few pathogens. This perspective changed with the advent of high throughput sequencing [4,17] and with recognition that the same bacteria that act as pathogens of mammals will sometimes also boost tick survival [11,12]. Ticks can also transmit more than one pathogen in a single bite, and thus it is informative to consider the interactions that contribute to a pathobiome [18,19]. Taxonomic profiling of bacteria is now generally based on PCR with universal primers for a conserved marker gene, followed by high-throughput sequencing of the amplicon. Although amplicon sequencing has biases, it is cost-effective, is supported by well-curated databases, and has a large foundation of previous studies for comparisons [4,19]. On the other hand, targeted identification of particular bacteria using specific primers and quantitative real time PCR (qPCR) can provide a fast and accurate assay of the target bacteria [20,21], although it provides little information about microbial community composition. Both approaches are multistep processes affected by variation in sample storage, extraction methods, primer specificity, number of PCR cycles, and data analysis. In a public health laboratory, all of these factors can cause significant constraints, and thus it can be helpful to apply both approaches to profiling microbiomes [22,23].

*Ixodes* ticks have recently expanded their range in Canada [24,25], which is a serious concern because they transmit *Borrelia* species that cause Lyme disease, the most common arthropod-vectored disease in North America [24,26,27]. The main vector of Lyme disease in North America is the blacklegged tick, *Ixodes scapularis*, the microbiome of which is dominated by *Rickettsia* in established populations, while more than 50% of ticks may also be infected with *Borrelia* in some areas [28,29]. Few microbiome studies of *I. scapularis* are available from areas where the tick is expanding its range or is adventitious, such as Alberta [30,31]. Adventitious ticks are defined in Canada as being found sporadically in an area that does not have multiyear reproducing populations [32]. Currently, *I. scapularis* ticks are considered to be established in five provinces but not in Alberta [33], and local public health messaging is influenced by whether ticks are regarded as being adventitious or from an established population [34]. Infection status of ticks as *Borrelia*-positive or negative is also critical to medical diagnoses [35]. Concern over exposure to Lyme disease has led to several surveys of *Borrelia burgdorferi* in *Ixodes* ticks [36,37], including a surveillance program established by Alberta in 2007 [38]. In Alberta, *Ixodes* ticks have been evaluated using qPCR for *B. burgdorferi*, with a yearly range of 10–19% testing positive during 2013–2018 [39]. However, little else is published about the microbiome of the ticks that potentially vector Lyme disease in western Canada.

Our study has three aims: (1) to characterize the bacterial microbiome of *Ixodes scapularis* ticks that are potential vectors of Lyme disease in Alberta, (2) to determine whether the composition of the microbiome of *Ixodes* ticks sampled in Alberta depends on their *Borrelia* infection status, and (3) to compare the performance of qPCR with 16S rRNA amplicon sequencing for detection of *Borrelia*. We used DNA extracts from a previous qPCR-targeted study on Lyme disease [39] and found that the microbiome of *I. scapularis* from Alberta was typical of that described for other regions. We also showed greater microbiome diversity in *Borrelia*-positive ticks, and strong correspondence between methods for detection of *Borrelia*. Our results expand understanding of tick-borne pathogens and establish a basis for future comparisons of *Ixodes* microbial assemblages in an area where *I. scapularis* is not yet established, but putatively adventitious ticks are commonly found. 

## 2. Materials and Methods 

### 2.1. Tick Specimens

We sampled the microbiome of 20 female *Ixodes* ticks, including 18 *Ixodes scapularis* and 2 identified as *Ixodes* sp. (Table 1), which were submitted during January to May 2016 to the Alberta Tick Surveillance Program (ATSP) of Alberta Agriculture and Forestry (ATSP) [40]. These ticks were removed from dogs or cats, then identified to life stage and species using the keys of Kierans and Clifford [41]. The two unidentified *Ixodes* sp. could not be identified to species level due to morphological damage to the samples. DNA was extracted from ticks using a QIAmp DNA Mini Kit (catalog no. 51304) without prior washing of the exterior of the tick. Extracts were stored at −80 °C in ATSP freezers until transfer to −70 °C at the University of Alberta. 

### 2.2. Control Samples

To provide context and calibration for our tick samples, we analyzed 3 additional control samples at the same time. First, we used a mock community (MOC) to estimate the lower limit of detection and to assess primer amplification biases. The MOC was provided by BEI Resources (Manassas, VA, USA; staggered low concentration HM783D, lot 60304010), with varied numbers of 20 bacterial strains in 17 different bacterial genera at concentrations ranging from 10^3^ to 10^6^ operons (= 16S rRNA sequence copies) per bacterial strain (Table S1). An aliquot of 2 µL of the total 35 µL was used in the initial 16S amplification of the MOC. Second, a sham extraction control (SEC) was used to estimate the amount and identity of bacteria associated with the extraction kit as well as the potential for aerosol contamination during sample handling. This SEC used the QIAmp DNA Minikit and followed all steps for extraction of a tick sample without using an input specimen, thus sampling DNA from extraction reagents and aerosols produced during the sham extraction process (Table S2). Third, a no-template PCR control (NTC) from the 16S amplifications was included to detect potential contaminants arising solely during the 16S amplification process (Table S3). 

### 2.3. Borrelia qPCR

Testing at ATSP established that 10 extracted tick samples were *Borrelia*-positive (B +) and 10 were *Borrelia*-negative (B−). The assay was based on a modified qPCR protocol that amplified the outer surface protein A (OspA) gene of *B. burgdorferi* directly without first screening for *B. burgdorferi* using the 23S marker, but was otherwise comparable to the method used by the National Microbiology Lab of Canada [42] with the following changes: the qPCR amplification was set for 50 cycles of 95 °C for 5 s and 61 °C for 10 s and 72 °C for 15 s. In 2016, a total of 215 *Ixodes* were tested from companion animals, of which 18% were positive for *B. burgdorferi* [39]. Positivity was based on qPCR using primers specific for *B. burgdorferi* OspA, including OspA forward: CTGGGGAAGTTTCAGTTGAAC, OspA reverse: TTGGTGCCATTTGAGTCGTA and OspA Probe: FAM-MGBCTGCAGCTTGGAATTCAGGCACTT [42].

### 2.4. 16S rRNA Sequencing

End point PCR amplification of the 16S gene encompassed 6 variable regions separately: V2, V3, V4, V6-7, V8, and V9 [43]. Amplification was conducted in 2 multiplex pools using the Ion 16S Metagenomics kit [44], with 25 amplification cycles of 95 °C for 30 s and 58 °C for 30 s. The first pool included V2, V4, and V8 and the second pool was V3, V6-7, and V9. Estimated length of amplicons ranged from 215 to 295 bp, depending on the region. Amplicons were visualized on an agarose gel to confirm amplification and quantified using Qubit High Sensitivity Assay. Aggregate amplicons for each of the 10 B+ tick samples and 10 B− samples were used for library preparation with 400 bp chemistry. Library concentrations were assessed with Agilent High Sensitivity Bioanalyzer and diluted to 26 pM before pooling. Sequencing used Ion Personal Genome Machine PGM™ with a 318C chip. To assess the repeatability of microbiome community proportions, we performed a second library preparation and sequencing run for the 5 qPCR B+ samples that had the lowest read counts in the first sequencing run. Sequences are deposited in National Center for Biotechnology Information NCBI’s Sequence Read Archive as BioProject Accession Number PRJNA668181.

### 2.5. Sequence Data Processing

Reads were processed using Ion Reporter Software 5.14.1.0, with sequences being retained for taxonomic assignment if both forward and reverse primers were present. A retention threshold of 10 identical copies per sequence was the default used in Ion Reporter and is intended to filter out reads with sequencing error or index switching. Taxonomic assignment was performed first by NCBI megaBLAST searches of the curated MicroSeq Reference Library v2013, followed by a second stage BLAST search of the curated Greengenes v13.5 database for all sequences that had an E-value greater than 0.01 in searches against the MicroSeq database. 

Counts for each taxon were additive for each variable region, e.g., a taxon detected by primers for V2 was also counted when detected in region V3. However, taxa could be classified at the level of family-only for some variable regions and the level of genus in the same or other variable regions. Designation as family-only could be due to the diagnostic limitations of a given variable region [45] or may result from natural variation or sequencing error in the sequences used to compare to a particular database. A single genus, for example *Streptococcus*, may be represented at the family-only level for some reads in region V6-7 and genus level for regions V2, V3, V4, V6-7, and V8. The total number of reads for *Streptococcus* species in the MOC sample were therefore spread over the level of Streptococcaceae family-only (15 reads) and the genus level of *Streptococcus* (9450 reads) (Table S4). 

Some variation in names is due to similarity of sequences to different databases that use different names for the same bacteria. For example, multistage BLAST searches for *Escherichia* sequence in the mock community resulted in most reads being assigned as *Escherichia/Shigella* while a minority of sequences were named *Escherichia*, on the basis of e-values larger than 0.01 when queried against the MicroSeq database but with similarity to the second stage Greengenes database at an e-value of less than 0.01 (Tables S1 and S4). The proportion of sequences expected for each bacterial genus is based on the number of operons provided in the mock community. 

Once reads were assigned taxonomically, the community matrix of sequence identifications produced by Ion Reporter was imported into an Excel spreadsheet, where reads present in the SEC sample or NTC sample were subtracted from the number of reads for each taxon. For taxa found in both SEC and NTC, the greater number of reads in either the SEC or NTC was subtracted from the sample numbers. Negative numbers were reset to zero. The community matrix was next imported into vegan 2.5–6 [46] and rarefied to 33,000 reads, which was the size of the smallest sample, rounded down (Figure S1). 

### 2.6. Diversity Measures and Associations with Tck Vriables

We used Pielou evenness and the Hill Number series H0-H3 as microbiome diversity measures. H0 is richness (number of species/taxa), while H1 corresponds to the exponential of Shannon entropy, and H2 is the inverse of Simpson’s index, with successive Hill numbers putting decreasing emphasis on rare members of a community. To assess beta diversity, we calculated Bray Curtis Distance measures and metaMDS using vegan 2.5–6.

A series of generalized linear models (GLM) were used to test the significance of associations between diversity measures (H0-H3, Pielou evenness) and several tick variables (*Borrelia* OspA status, host animal, date collected, travel history outside the province in the previous 2 weeks). Pielou evenness was modelled using a beta distribution as values are bounded between 0–1. For Pielou evenness, we used B+ status and host animal as model variables. H0 (richness expressed as count data) and H1–H3 (continuous integers) were modelled using negative binomial and gamma distributions, respectively, each with a log-link function. Categorical variables were *Borrelia* OspA status (negative/positive), host animal (dog or cat), and travel history outside province in last 2 weeks (yes/no/not available), while date collected (expressed as Julian date) was continuous. To compare the 2 sequencing runs, we ran a series of GLMs using only the run number as a variable. Beta distribution was used for Pielou evenness, negative binomial for H0, and gamma distributions for H1-H3.

## 3. Results

### 3.1. Ixodes Scapularis Microbiome Characteristics 

We sampled the microbiome of 20 female ticks that were previously PCR-tested as *Borrelia*-positive (B+) or *Borrelia*-negative (B−) (Table 1). The 20 Alberta *Ixodes* ticks gave a mean of 112,676 (Standard Error = SE ± 5742) total raw 16S reads before filtering per tick in the first sequencing run (Table 1), with a mean of 64,377 (SE ± 3058) remaining after filtering out all reads that were not represented by at least 10 identical sequence copies. We sampled five B+ ticks to greater depth in a second 16S rRNA sequencing run. Finally, we sequenced three controls, a mock bacterial community, a sham extraction, and a no-template control. 

**Figure 1:**
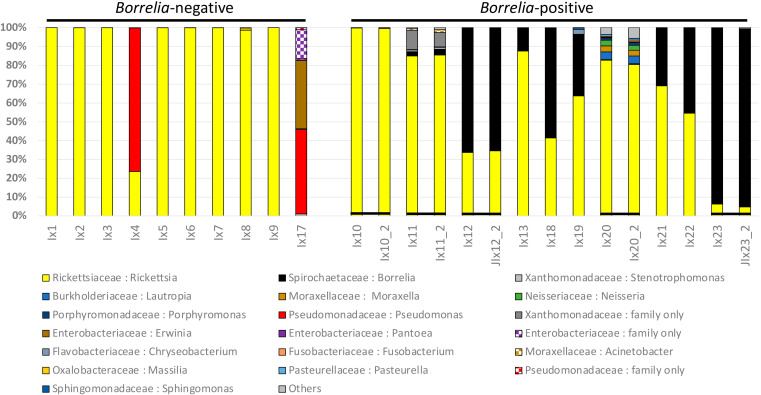
Proportions of bacterial reads detected for 10 *Borrelia*-negative and 10 *Borrelia*-positive ticks. Bacterial taxa found at greater than 1% of any single tick are distinguished; the remaining lower frequency taxa are grouped as “Other”. Repeated sampling of five ticks is shown with black bars joining the same samples.

We showed the proportions of bacterial reads assigned to each bacterial taxon for B− and B+ ticks (Figure 1). Bacterial taxa that comprised more than 1% of the total reads in at least one tick are shown with all taxa consistently representing less than 1% of reads grouped as “Other”. *Rickettsia* were generally the most common bacteria in both B− and B+ tick samples (Figure 1), comprising a mean of 82% in the B− and 62% in the B+ groups. *Rickettsia* comprised > 50% of reads in 8 of 10 B− and 7 of 10 B+ ticks, with proportions varying from 100% to 0.05%. Only two B− ticks had less than 99% *Rickettsia.* For these two ticks*, Pseudomonas* comprised 76% (tick I × 4) and 45% (I × 17) of the total reads. The I × 17 tick also contained 53% Enterobacteriaceae (including 36% *Erwinia*). 

We related the presence of reads corresponding to *Borrelia* to the results of the qPCR test. No *Borrelia* reads were found in any B− ticks, whereas *Borrelia* comprised the second most common bacterial taxon in B+ ticks (Figure 1). *Borrelia* proportions in B+ ticks ranged from 93% to 0%, after reads with less than 10 identical copies were filtered out. One qPCR-positive tick (I × 20) had 0% *Borrelia* reads for 16S testing when filtered at a 10-copy threshold, but nonetheless had 21 *Borrelia* reads when a single copy filtering threshold was used. I × 20 also had small numbers of other Spirochaetaceae reads at the single copy threshold (15 *Spirochaeta*, 70 *Treponema*, and 1 family level only, totaling 107 Spirochaetaceae reads with the 21 *Borrelia*) that were not found in any other tick samples. This I × 20 tick had the largest number of bacterial genera (28 total in 10-copy filtered sequences). After *Borrelia*, the most frequent bacterial taxon was Xanthomonadaceae, which was only represented by about 10% of the reads in a single tick (I × 11, a B+ tick).

To assess the repeatability of microbiome surveys of B+ ticks, we sequenced five B+ samples with the lowest number of reads again after a second library preparation that gave an increase in reads (mean = 318,783 raw reads per sample; 216,558 reads after 10-copy filtering). The proportions of reads for major bacterial groups remained essentially identical after the second preparation (Figure 1). Sample I × 20 produced 98 *Borrelia* raw reads in the second run, which became 12 reads after filtering at the 10-copy threshold. This tick also had correspondingly greater numbers of other Spirochaetaceae. These additional Spirochaetaceae taxa remained undetected in the four other samples included in the second run.

### 3.2. Comparison of Microbiomes With or Without Borrelia

**Figure 2:**
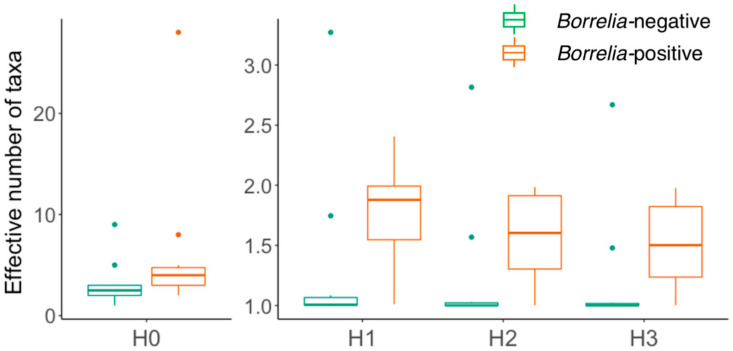
Diversity of bacteria in *Borrelia*-negative and *Borrelia*-positive ticks represented by Hill numbers: H0 (richness), H1 (exponential of Shannon entropy), H2 (inverse of Simpson’s index), and H3.

To assess the diversity of the microbiomes of B+ and B− ticks, we represented the number of taxa by Hill numbers (Figure 2). The B+ ticks had noticeably greater overall bacterial diversity than ticks without *Borrelia*. In terms of taxon richness (H0), B+ ticks were twice as diverse, with an average of 6.3 ± 2.5 bacterial taxa detected compared to B− ticks that only had an average of 3.1 ± 0.75 (*p* < 0.014, Table S5). This trend continued for H1-H3, with B+ ticks having 1.8 to 1.5 “effective taxa” (in this case mainly genera) compared to B− ticks with 1.3-1.2.

We visualized the differences in community composition between B+ and B− ticks using nonmetric multi-dimensional scaling (NMDS) of Bray–Curtis distance dissimilarity measures. An ordination plot shows that the diversity of 8 of 10 B− ticks was contained within the greater range of values for B+ ticks (Figure S2A). The two B− tick exceptions (I × 4 and I × 17) had very low *Rickettsia* proportions. The data were well represented in two dimensions with stress values less than 0.01 and *R^2^* of 1 (Figure S2B).

To test for associations between diversity and sample characteristics, we used generalized linear models (GLMs). Several variables did not have significant associations with any diversity measures, including the mammalian host of the tick (dog vs. cat), collection date, and travel history in the previous 2 weeks (Table S5). However, B+/B− status was significantly associated with all diversity measures, including Pielou evenness and the Hill numbers H0-H3 (Table S6). 

To determine whether microbiome sequencing depth changes the proportions of taxa identified, we compared the data from the first and second sequencing runs. Although the second sequencing run of five tick samples provided 3.4 times more reads than the first, there were no significant differences in diversity between the two sequencing runs (Figure 1, Table S6). The number of bacterial taxa detected (H0) in the second run was numerically greater (16.4 ± 10.4 effective number of taxa) when compared to the same five samples in the first run (9.4 ± 4.7), but the difference was not statistically significant. Since H1-H3 have greater weighting on the most abundant taxa in samples, these diversity measures are even less likely to demonstrate differences. No measures were significantly different between the two runs, and hence assessment of tick microbiomes was robust to sequencing depth (Figure 2, Table S6).

### 3.3. Borrelia Detection: 16S Versus qPCR

To assess the sensitivity of the qPCR test compared to microbiome sequencing, we examined the sequences that corresponded to *Borrelia*. Characterization of the microbiome of ticks using 16S surveys was congruent with the prior determination of *Borrelia* status on the basis of qPCR. Sequences from the first 16S library preparation had no *Borrelia* in any of the 10 qPCR B− samples, while showing *Borrelia* in 9 of the 10 B+ samples. This first set of sequences showed no *Borrelia burgdorferi* in one B+ tick (I × 20) and only 13 *Borrelia* reads in a second sample (I × 10). These two samples were among the five samples in the second library preparation, which had an increase by a factor of 3.4X in the average number of reads both before and after reads were filtered and taxonomically classified. The presence of *Borrelia* in the tick sample was more evident in the second run, confirming the qPCR results for I × 20. Across all samples that were *Borrelia*-positive, four of the six 16S variable regions produced at least some reads (Table S7). Within each sample, the largest number of *Borrelia* reads per sample was produced by primers for variable region V4 (7 of 15 samples) or region V6-7 (5 of 15), while regions V8 and V9 were consistently the worst or had no reads at all. In addition to confirming the qPCR-based *Borrelia* status of all ticks, 16S-based methods showed that all 20 tick specimens contained *Rickettsia*, a bacterial genus with potential pathogenicity to mammals that in this case may represent a nutritional symbiont of the tick [47]. No other bacterial pathogens of mammals vectored by ticks in Canada [24], including *Ehrlichia*, *Anaplasma*, or *Francisella,* were detected in our samples. 

To estimate the limits of detection of different bacterial taxa, we included three control samples in our microbiome sequencing. To assess the efficacy of our primers for amplifying different taxa of bacteria, we sequenced a mock bacterial community (MOC) composed of known bacteria in known proportions. MOC results showed both overestimates (e.g., *Escherichia/Shigella*) and underestimates (e.g., *Staphylococcus*) of the true proportions of reads for different taxa in the mock community, compared to the expected number of reads (Table S1). We detected all members of the mock community that had greater than 10^4^ operons (16S copies) per bacterial strain in the mock community, with the sole exception of *Listeria*. Utilizing a minimum filtering threshold of 10 identical sequences and both primers, we detected no members of the MOC with fewer than 10^3^ operons. To examine the efficacy of our primers to detect different taxa, we assigned reads to each 16S rRNA variable region that was amplified (Table S4). 

To assess the reads arising from the sequencing environment, we sequenced a sham extraction control. The sham extraction control (SEC) produced 71,920 reads, of which 30,830 reads had primers detected on each end and were mapped to the taxonomic database (Table S2). Finally, we included a no template control (NTC) to assess the reads arising from the amplification steps. The NTC control gave a total of 44,610 reads, of which 19,515 reads had both ends and were mapped to the taxonomic database (Table S3). Taxa associated with the SEC and NTC were primarily taxa described as being associated with PCR and extraction kit reagents (e.g., *Pseudomonas* [48,49]); however, there were 18 reads associated with *Rickettsia* in the kit control. Using a threshold value of a single copy, we detected 25 *Rickettsia* reads and 2 *Borrelia* reads in the MOC. This is within the range expected for index switching (0.167%) with PGM [50].

## 4. Discussion

The microbiome of *Ixodes scapularis* ticks in Alberta is dominated by *Rickettsia* endosymbionts, with *Pseudomonas* more common in some individuals, making it similar to the microbiome composition reported for these ticks in established areas of their range [28,29,51]. Alberta ticks that carried *Borrelia* had significantly greater microbiome diversity than ticks that had no *Borrelia*, a finding that suggests an altered microbiome ecology in *Borrelia*-positive ticks, as well as the potential to use microbiome diversity as an indicator of elevated risk of infection by *Borrelia*. Depending on filtering thresholds, 16S rRNA marker gene amplification and scanning was less sensitive for detection of *Borrelia* than was qPCR, leading us to suggest that qPCR is the preferred method of detection when there are known pathogens in a region. However, the generally elevated bacterial diversity found in *Borrelia*-positive ticks suggests that the current focus on a single predetermined pathogen could lead to significant biases in understanding the interactions and disease potential of bacteria associated with *I. scapularis* in Alberta.

### 4.1. Components of the Microbiome

Our finding that the microbiome of *I. scapularis* in Alberta resembles the microbiome of this species elsewhere in the range of the tick gives a starting point for better identification of its components. Ogden et al. [52] found the *Borrelia* sequence type in a single positive tick from Alberta was most closely related to *Borrelia* found in Manitoba and the midwestern USA. *Rickettsia* sequences in Alberta ticks likely represent a known *I. scapularis* endosymbiont, *Rickettsia buchneri,* which was described from an isolate in Minnesota, USA [47]. However, it is not feasible to reliably distinguish among *Rickettsia* species in our data [53], as the short 16S rRNA fragments that are widely used to survey bacteria do not accurately identify bacteria to the species level [54,55]. Standard gene-based identifications at the species level in *Rickettsia* require four protein coding genes (gltA, ompA, ompB, and gene D) in combination with 16S rRNA gene sequences [56]. Since other *Rickettsia* species such as *R. rickettsii* that are pathogenic to mammals may also be found in *I. scapularis* [2], it would be appropriate to test for these *Rickettsia* using nested PCR or qPCR with species-specific primers. 

Although *Rickettsia* were the most abundant bacteria in most of the ticks we sampled, some ticks were dominated by *Pseudomonas*, various Enterobacteriaceae, or *Borrelia*. Two *Borrelia*-negative ticks had large proportions of *Pseudomonas*, which has also been found in blacklegged ticks in Ontario, Canada [43,57], and eastern USA [51,58]. The role of *Pseudomonas* in the microbiome of ticks remains to be clarified, although a study focused on *Pseudomonas* from healthy vertebrates in Spain recovered *Pseudomonas fluorescens* (11 isolates) and *Pseudomonas gessardii* (6 isolates*)* from two ticks that were included in their sampling [59]. *Pseudomonas fluorescens* can have antibiotic and probiotic properties and is part of a healthy soil community [60], while *P. gessardii* is a member of the same *P. fluorescens* group but was isolated from mineral waters rather than soil [61]. Thus, the presence of these bacteria in ticks is most likely an indication of the ubiquity of *Pseudomonas* in a variety of habitats [62]. The abundance of *Pseudomonas* relative to *Rickettsia* in our 16S rRNA dataset may also partly reflect the greater number of copies of 16S in *Pseudomonas.* This genus has an average of 4.9 copies of the 16S gene per genome (range 1–9, mode = 4), while *Rickettsia* averages 2.1 (range 1–6, mode = 1: rrnDB version 5.6 [63]). However, as recommended by Louca et al. [64], in order to retain comparability among studies, we made no corrections for 16S rRNA gene copy number to our dataset. 

One of the two *Borrelia*-negative ticks with few *Rickettsia* (I × 17) had 36% of its reads assigned to *Erwinia,* a member of the Enterobacteriaceae. The presence of large amounts of *Erwinia*, a genus best known for plant pathogens like fireblight [65], also suggests the possibility of a tick pathogen. *Erwinia aphidicola* is a pathogen of the pea aphid [66], while other species of *Erwinia* are associated with diverse arthropods such as bark beetles [67] and thrips [68], although their roles in these insects are unknown. In addition to *Erwinia*, 15% of the sequences assigned to I × 17 could only be identified as Enterobacteriaceae at the family level. Undescribed Enterobacteriaceae were the most common bacteria found in *Ixodes scapularis* in North Carolina and may be an important commensal of arthropods [51]. In addition to being present in ticks in our current study, bacteria that could only be identified as bacterial family Enterobacteriaceae have been reported in *I. scapularis* from Ontario and *Ixodes angustus* in British Columbia (BC) [43]. 

Tick species are often distinguished from each other by characteristic assemblages of bacteria [4,17,69]. This phenomenon can be used to support identification of a tick that is no longer morphologically intact or whose DNA has been used up, as was the case for our samples. On this basis, the most likely species identity of the two ticks labelled as *Ixodes* sp. (I × 20, I × 23) is *I. scapularis*. In addition to *I. scapularis*, two other *Ixodes* are plausible for samples in the Alberta ATSP survey: *Ixodes angustus* and *Ixodes pacificus*. The microbiome of *I. angustus* is normally dominated by *Francisella*-like endosymbionts [43], which were not found in the two *Ixodes* sp. samples. Alternatively, these samples could have been *I. pacificus*, but this species is typically only found in Alberta on animals that have recently travelled to BC, which was not true of these two ticks. Further surveys of geographic variation in the composition of the microbiome of *Ixodes* ticks, both between and within species, would be highly informative in establishing the potential source regions of ticks in Alberta, especially since *I. scapularis* is currently regarded as being solely of adventitious origin in this region [30,70].

### 4.2. Borrelia Infection Status and Tick Microbiome Diversity

We found that *Borrelia*-positive adult female ticks had significantly greater overall bacterial diversity than those that were *Borrelia*-negative. This result is consistent with the finding by Narasimhan et al. [71] that laboratory-reared *I. scapularis* ticks with greater microbiome diversity were more likely to be successfully colonized by *B. burgdorferi.* These ticks also had more intact and thicker peritrophic membranes, which may protect *B. burgdorferi* from digestion by the tick [71,72]. In the Narasimhan et al. [71] study, tick dysbiosis (=altered microbiome composition), rather than absolute bacterial diversity, was considered to be the key factor in colonization success. In fact, the microbiome diversity of the gut of ticks may be quite low, according to work by Ross et al. [69], and microbiome diversity can differ greatly between specific tissues and whole ticks [17].

In contrast to our results, three recent studies have shown no significant difference in bacterial diversity associated with *Borrelia* infection status [58,73,74], and a fourth study was ambiguous [28]. However, one of these studies reported results for a mix of females and males [73], which would have increased the variance in an already noisy system [17]. A second study [74] analyzed nymphs of *Ixodes pacificu*s and found no significant differences in richness or evenness. A third study [58] distinguished males from females and found no significant differences in bacterial community composition between B+ and B− ticks, on the basis of unweighted UniFrac distance; however, they did not address differences in numbers of bacterial taxa for B+ and B− ticks. Finally, the fourth study [28] gave mixed results. Richness in B+ ticks was significantly higher when all life stages were considered; however, there was no difference in diversity between B+ and B− nymphal ticks alone [28]. This variability in results underscores the need to control for life history stages when evaluating the significance of patterns relating to *Borrelia* status.

One life history factor in ticks that is associated with *Borrelia* infection is overwintering success. In Switzerland, *Ixodes ricinus* ticks infected by *Borrelia* are more likely to overwinter successfully [12]. If such ticks also have increased bacterial diversity, then this may provide increased opportunities for functional adaptations, in keeping with observations in other organisms that microbiome diversity is generally beneficial [75].

The source of the blood meal of a tick is another ecological factor that can impact its microbiome diversity. For example, deer blood has bacteriolytic effects on *Borrelia* in *I. scapularis* [76]. In fact, Brinkerhoff et al. [28] have proposed that this phenomenon should cause reduced overall bacterial diversity across the microbiome of fed ticks. Swei and Kwan [77] also reported reduced microbiome diversity of *I. pacificus* after feeding on western fence lizards, which are known to be refractory to *Borrelia* [78,79]. However, the *I. scapularis* ticks sampled in our study were all feeding on dogs and cats, which are not considered refractory to *Borrelia.* Furthermore, Thapa et al. [58] reported that ticks feeding on dogs had increased bacterial diversity compared to unfed ticks, although *Borrelia* was not detected in any of their ticks. Thus, our finding of increased bacterial diversity in the ticks that are infected with *Borrelia* will need to be reexamined with larger sample sizes, ticks of both sexes and all life stages, and in ticks with a range of feeding statuses. However, if this result is confirmed, it would raise the question of whether there is an elevated risk of co-transmission of other bacterial pathogens by ticks that are carrying *Borrelia*. 

A variety of studies have shown that the infection status of *Ixodes* ticks by *B. burgdorferi* is affected by the presence of other co-circulating pathogens [80,81,82]. Positive associations have been found between *B. burgdorferi* and *Babesia microti* [80,81]*,* while mixed positive and negative associations are reported for *Anaplasma phagocytophilum* [81,82]. This may be explained by differences in the role of the tick peritrophic membrane in facilitating colonization by different bacteria. In contrast to *B. burgdorferi*, which benefits from the protection provided by an intact peritrophic membrane [71], *A. phagocytophilum* is better able to colonize ticks with a more permeable peritrophic membrane [83]. This contrast suggests that *B. burgdorferi* and *A. phagocytophilum* should rarely be found as co-infections in the same tick. However, this expectation is contradicted by the study of Pokutnaya et al. [81], who reported that female ticks were more likely to be infected with *B. burgdorferi* if they are also infected with *A. phagocytophilum*. The interactions among these bacteria are still poorly understood, and it will ultimately also be necessary to determine whether such co-infected ticks have a reduced probability of actually transmitting either *A. phagocytophilum* or *B. burgdorferi*.

Although no *A. phagocytophilum* was detected in the 20 ticks examined in our study, this sample size is insufficient to conclude that *A. phagocytophilum* is not present in Alberta. On the basis of increasing detection of *A. phagocytophilum* in ticks in Ontario [84], we recommend including surveillance of *A. phagocytophilum* in tick surveys in Alberta. At least one human case with no history of travel has been detected in Alberta, which suggests that *A. phagocytophilium*-infected ticks may be found in Alberta sporadically [85]. A multiplex qPCR protocol for both *B. burgdorferi* and *A. phagocytophilium* has already been developed at the Canadian National Microbiology Laboratory and could be easily incorporated into the Alberta tick surveillance program [42].

### 4.3. Effectiveness of 16S rRNA Surveys

Our study found that *B. burgdorferi* was detected consistently in the same tick samples using both qPCR and 16S rRNA end point detection, although this finding was dependent on 16S sequencing depth at the lowest levels. Targeted qPCR detection of specific pathogens can be more cost-efficient than broad-range primers, and qPCR primers can be designed for higher annealing temperatures and therefore give greater specificity than general PCR primers that may include degenerate bases [86]. Competition for template and GC bias can also mask low levels of a pathogen to a greater degree in general 16S amplification than with qPCR [87]. In our study, the PCR annealing temperatures for qPCR and 16S rRNA end point detection differed by only 2 °C, and thus competition for template and the degenerate primers used in 16S amplification are more likely explanations for the more decisive detection by qPCR. 

Microbiome scans using 16S rRNA are widely regarded as producing inherently noisy datasets, in part due to sequencing platforms with different error profiles [4,88]. For example, Ion PGM sequencing has a greater number of indel errors than Illumina sequencing [89,90]. To reduce any sequencing error, a threshold number of identical sequences is considered necessary, on the assumption that errors will be random along the sequence and deeper coverage will allow sequencing errors to be distinguished from biological variation [91]. In order to filter such errors, Ion PGM software uses a default number of 10 identical sequences for taxonomic profiling; if fewer than 10 copies of a specific sequence are found then these all remain unclassified. A threshold of 10 reads may generally provide a workable balance between accuracy and inclusion, but it risks overlooking rare variants. For example, when sample I × 20 data was analyzed with a threshold level of a single read, the first sequencing run resulted in 107 sequences matching Spirochaetaceae at the family level. However, no particular single sequence was found to have more than 10 copies and therefore the sample was considered to be *Borrelia*-negative in the initial analysis. Smaller thresholds for read filtering may be more appropriate for Illumina sequencing [88], but the most appropriate copy number for filtering has not yet been established for any high throughput sequencing platform [92].

Control samples provide a crucial test of the likelihood of technical artifacts in the detection of taxa reported in microbiome surveys, and their use is now expected in microbiome studies [92,93]. Predefined “mock communities” are one such positive control for PCR amplification in 16S rRNA scans [93]. Our sequencing results for the staggered mock community show that different genera are detected at differing abundances. This may be due to biases in primers, GC content affecting gene amplification and sequencing as well as classification [92,94]. In our MOC sample, no taxa were detected that had an expected relative abundance of 0.02%, while five of six taxa were detected at 0.22% and all taxa comprising at least 2% of the total sample were found (Table S1). Detection of low abundance bacteria (0.22% or below) was not related to GC content. Extrapolating from the mock community to our tick samples, we should have detected most of the taxa present in our tick samples if they comprised at least 0.22% of the 16S sequences in the total sample. 

Acting as a complement to the MOC, extraction controls (SEC) and no-template controls (NTC) commonly identify a number of taxa that may be erroneously attributed to a biological sample [48]. The number and diversity of reads that we found in both the SEC and NTC indicates that much of the contamination in 16S sequencing occurred at the stage of DNA extraction. This is most clearly demonstrated by the presence of 18 reads attributed to *Rickettsia* in the SEC and no reads attributed to *Rickettsia* in the NTC. The positive (MOC) and negative (SEC, NTC) standardized controls that we included in our experimental design allowed us to determine where possible contamination arises as well as to pinpoint reagent-associated contaminants for subtraction from biological samples. This should also facilitate comparisons to similar studies in other labs. 

### 4.4. Variation in Tick Microbiomes as a Tool for Monitoring Disease Risk

The microbiome of ticks can have substantial geographic variation [51]. *Ixodes scapularis* apparently does not have established, reproducing populations in Alberta [30,70], and thus the ticks found in this region are assumed to have been derived via recent, continuing dispersal from populations of ticks that are established in Manitoba, eastern Canada, or adjacent areas of the United States south to Mexico [70]. This raises the question of whether microbiome variation in *I. scapularis* could be used to identify the geographic origins of the *I. scapularis* ticks that are found in Alberta. 

The presence of *I. scapularis* in Alberta is most likely due to tick attachments to migratory birds, and potentially also to deer and other mobile mammals, which allow dispersal well beyond the established range of the tick [70,95]. Such dispersing ticks are expected to bring an assemblage of bacteria that resembles their original habitats, in addition to maternally transmitted bacteria associated with the tick species and bacteria picked up from exposure to earlier hosts. It may therefore be possible to explain the presence and origin of potential pathogens in a geographic region—such as Alberta—by building a database of the bacteria associated with ticks from different areas outside the region of interest. Such a catalog of bacteria would also advance our understanding of the spread of tick-borne pathogens, expand our understanding of tick biology and provide an opportunity to clarify hypotheses relating to microbial interactions of the tick microbiome. Our study provides a starting point for monitoring incursions of blacklegged ticks and their associated bacteria in concert with environmental changes. 

## Figures and Tables

**Table 1:** Tick collection data and control samples. All ticks were engorged adult females. Control samples: MOC (mock community), SEC (sample extract control), NTC (no-template control). *Outside Alberta* travel applies when host animals were known to have travelled outside Alberta within 2 weeks of collection. *Bb qPCR* status is the assay for *Borrelia burgdorferi* OspA gene. *16S reads* are the numbers of raw reads before filtering and taxonomic classification

Tick ID #	Date Collected	Outside Alberta	Tick Species	Host Animal	Bb qPCR	16S Reads-1st prep.	16S Reads-2nd prep.
I × 1	2016-05-06	Yes	*I. scapularis*	Dog	Neg.	91,317	-
I × 2	2016-05-02	No	*I. scapularis*	Cat	Neg.	71,209	-
I × 3	2016-05-13	Unkn.	*I. scapularis*	Cat	Neg.	134,766	-
I × 4	2016-05-17	No	*I. scapularis*	Dog	Neg.	109,864	-
I × 5	2016-05-18	No	*I. scapularis*	Cat	Neg.	145,698	-
I × 6	2016-05-19	No	*I. scapularis*	Dog	Neg.	81,204	-
I × 7	2016-05-20	No	*I. scapularis*	Dog	Neg.	62,927	-
I × 8	2016-05-25	No	*I. scapularis*	Dog	Neg.	104,139	-
I × 9	2016-05-26	Unkn.	*I. scapularis*	Dog	Neg.	110,758	-
I × 10	2016-01-17	Yes	*I. scapularis*	Dog	Pos.	118,427	289,781
I × 11	2016-04-11	No	*I. scapularis*	Dog	Pos.	130,881	339,121
I × 12	2016-04-18	No	*I. scapularis*	Dog	Pos.	100,333	321,740
I × 13	2016-04-27	No	*I. scapularis*	Dog	Pos.	104,545	-
I × 17	2016-05-17	No	*I. scapularis*	Dog	Neg.	174,362	-
I × 18	2016-05-08	No	*I. scapularis*	Cat	Pos.	120,645	-
I × 19	2016-05-13	No	*I. scapularis*	Dog	Pos.	123,266	-
I × 20	2016-05-31	No	Ixodes spp.	Dog	Pos.	126,995	347,373
I × 21	2016-04-28	No	*I. scapularis*	Dog	Pos.	98,205	-
I × 22	2016-05-20	No	*I. scapularis*	Dog	Pos.	127,989	-
I × 23	2016-05-04	Unkn.	*Ixodes* spp.	Dog	Pos.	115,995	295,902
MOC	n/a	n/a	n/a	n/a	n/a	95,023	-
SEC	n/a	n/a	n/a	n/a	n/a	71,920	-
NTC	n/a	n/a	n/a	n/a	n/a	44,610	-
Ticks$\bar{x}$						112,676.3	318,783.4
Ticks SE						5,741.7	11,411.4

Neg = negative, Pos = positive, Unkn = unknown.

## Supplementary Materials

The following are available online at https://www.mdpi.com/2414-6366/5/4/173/s1: Figure S1. Rarefaction curves for the bacterial microbiome of 20 *Ixodes* ticks. Figure S2. Non-metric multidimensional scaling (NMDS) ordination (A) and two-dimensional stress plot (B) of Bray–Curtis dissimilarities among microbiome communities of *Borrelia*-negative and *Borrelia*-positive ticks. Table S1. Composition of expected and observed bacterial taxa in the mock community (MOC). Table S2. Identity of bacterial taxa detected in the sham extraction (SEC) control. Table S3. Identity of bacterial taxa detected in the no template PCR (NTC) control. Table S4. Bacterial taxa detected in the mock community (MOC), subdivided by the number of reads for each 16S variable region. Table S5. Generalized linear model (GLM) outputs for associations among five bacterial diversity measures and five tick variables. Table S6. GLM outputs for tests of association within diversity measures for five B+ tick microbiomes sequenced at low read depth (mean = 64,377 after filtering) and higher read depth (mean = 216,558 after filtering). All comparisons use data rarefied to 33,000 reads. Table S7. Number of reads assigned to *Borrelia* (A) and ranking (B) by 16S variable region.
