# Supplementary material for: Microbiome Composition and Borrelia Detection in Ixodes scapularis Ticks at the Northwestern Edge of Their Range
Source: Trop Med Infect Dis. 2020 Nov 18;5(4):173. doi: 10.3390/tropicalmed5040173 (PMC7709646; doi:10.3390/tropicalmed5040173)
Supplement: Supplementary file 1 [file tropicalmed-05-00173-s001.zip › SupplFig2.pdf]

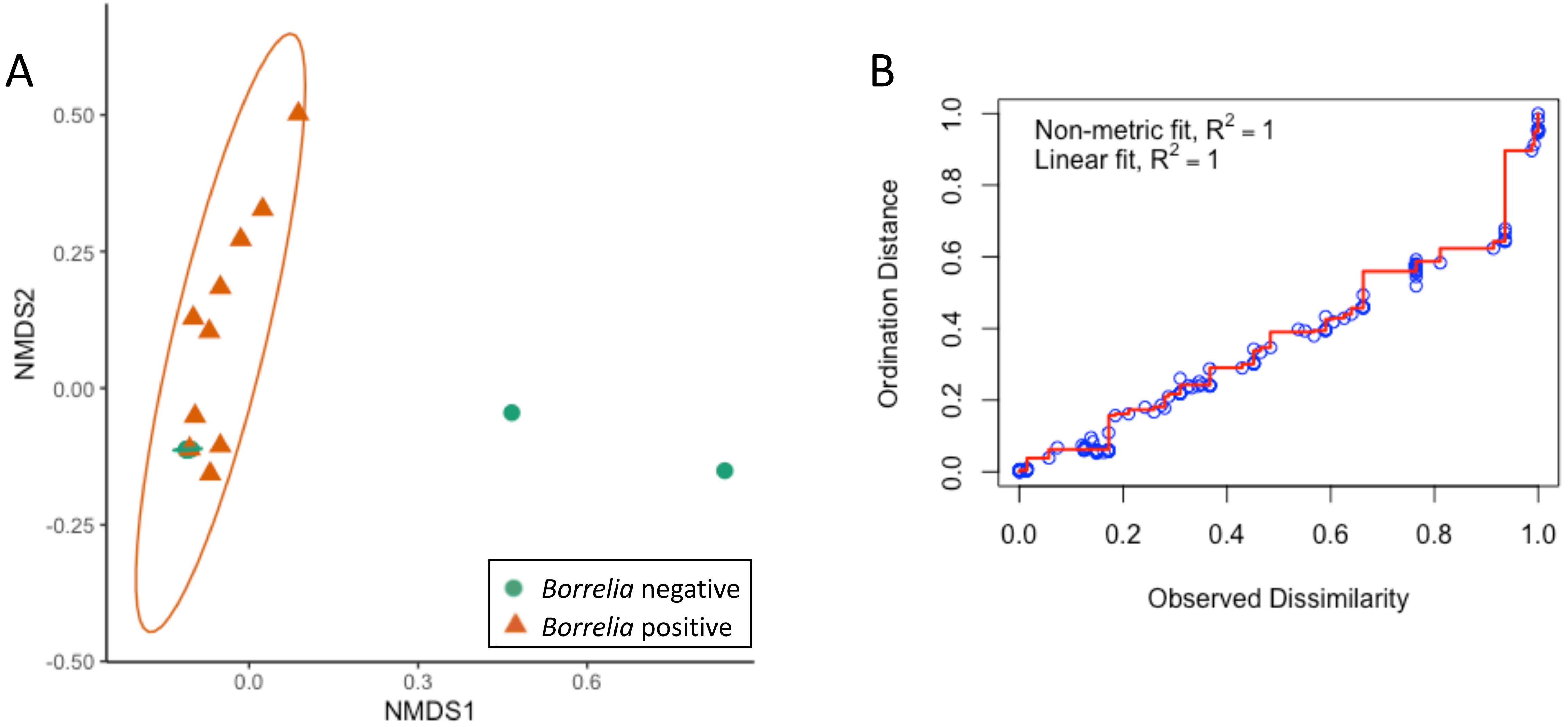

Suppl. Fig. 2. NMDS ordination (A) and 2-dimensional stress plot (B) of Bray Curtis dissimilarities among microbiome communities of *Borrelia*-negative and *Borrelia*-positive ticks. Ellipse in A is a multivariate t- distribution.
