# Supplementary material for: Microbiome Composition and Borrelia Detection in Ixodes scapularis Ticks at the Northwestern Edge of Their Range
Source: Trop Med Infect Dis. 2020 Nov 18;5(4):173. doi: 10.3390/tropicalmed5040173 (PMC7709646; doi:10.3390/tropicalmed5040173)
Supplement: Supplementary file 1 [file tropicalmed-05-00173-s001.zip › SupplFig1.pdf]

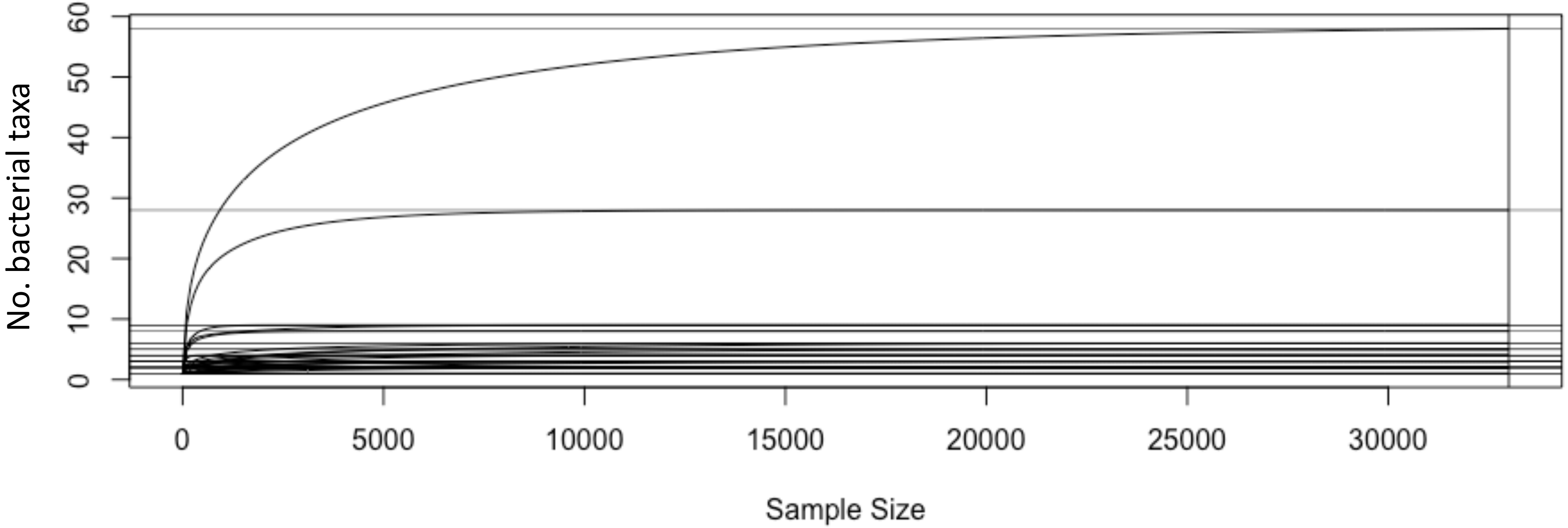

Supplementary Fig. 1. Rarefaction curves for the bacterial microbiome of 20 *Ixodes* ticks. Vertical line is at 33000 reads.
